# Crystal structure of the new palladium complexes tetra­kis­(1,3-di­methyl­imidazolium-2-yl­idene)palladium(II) hexa­deca­carbonyl­tetra­rhenium diethyl ether disolvate and octa-μ-carbonyl-di­carbonyl­tetra­kis­(tri­phenyl­phosphane)palladium­dirhenium (unknown solvate)

**DOI:** 10.1107/S2056989021009270

**Published:** 2021-09-14

**Authors:** Sergey Shapovalov, Olga Tikhonova, Ivan Skabitsky

**Affiliations:** a Kurnakov Institute of General and Inorganic Chemistry, 119991, Leninskii pr. 31, Moscow, Russian Federation

**Keywords:** palladium, rhenium, carbon­yl, NHC, tri­phenyl­phospine, crystal structure

## Abstract

The investigation of the coordination chemistry of heterometallic transition-metal complexes of palladium (Pd) and rhenium (Re) led to the isolation and crystallographic characterization of tetra­kis­(1,3-di­methyl­imidazolium-2-yl­idene)palladium(II) hexa­deca­carbonyl­tetra­rhenium diethyl ether disolvate, [Pd(C_5_H_8_N_2_)_4_][Re_4_(CO)_16_]·2C_4_H_10_O or [Pd(IMe)_4_][Re_4_(CO)_16_]·2C_4_H_10_O, (**1**), and octa-μ-carbonyl-di­carbonyl­tetra­kis­(tri­phenyl­phosphane)palladium­dirhenium, [Pd_4_Re_2_(C_18_H_15_P)_4_(CO)_10_] or Pd_4_Re_2_(PPh_3_)_4_(μ-CO)_8_(CO)_2_, (**2**), from the reaction of Pd(PPh_3_)_4_ with 1,3-di­methyl­imidazolium-2-carboxyl­ate and Re_2_(CO)_10_ in a toluene–aceto­nitrile mixture. In complex **1** the Re—Re bond lengths [2.9767 (3)–3.0133 (2) Å] are close to double the covalent Re radii (1.51 Å). The palladium–rhenium carbonyl cluster **2** has not been structurally characterized previously; the Pd—Re bond lengths [2.7582 (2)–2.7796 (2) Å] are about 0.1 Å shorter than the sum of the covalent Pd and Re radii (1.39 + 1.51 = 2.90 Å). One carbene ligand and a diethyl ether mol­ecule are disordered over two positions with occupancy ratios of 0.5:0.5 and 0.625 (15):0.375 (15) in **1**. An unidentified solvent is present in compound **2**. The given chemical formula and other crystal data do not take into account the unknown solvent mol­ecule(s). The SQUEEZE routine [Spek (2015 ▸). *Acta Cryst.* C**71**, 9–18] in *PLATON* was used to remove the contribution of the electron density in the solvent region from the intensity data and the solvent-free model was employed for the final refinement. The cavity with a volume of *ca* 311 Å^3^ contains approximately 98 electrons.

## Abstract 2

The investigation of the coordination chemistry of heterometallic transition-metal complexes of palladium (Pd) and rhenium (Re) led to the isolation and crystallographic characterization of tetra­kis­(1,3-di­methyl­imidazolium-2-yl­idene)palladium(II) hexa­deca­carbonyl­tetra­rhenium diethyl ether disolvate, [Pd(C_5_H_8_N_2_)_4_][Re_4_(CO)_16_]·2C_4_H_10_O or [Pd(IMe)_4_][Re_4_(CO)_16_]·2C_4_H_10_O, (**1**), and di­carbonyl­octa-μ-carbonyl-tetra­kis­(tri­phenyl­phosphane)palladiumdirhenium, [Pd_4_Re_2_(C_18_H_15_P)_4_(CO)_10_] or Pd_4_Re_2_(PPh_3_)_4_(μ-CO)_8_(CO)_2_, (**2**), from the reaction of Pd(PPh_3_)_4_ with 1,3-di­methyl­imidazolium-2-carboxyl­ate and Re_2_(CO)_10_ in a toluene–aceto­nitrile mixture.

## Chemical context   

Bimetallic catalysts comprising palladium (Pd) and rhenium (Re) have important applications in alkane reforming, industrial chemical production, hydro­dechlorination and biomass conversion (Thompson & Lamb, 2016 ▸; Bonarowska *et al.*, 1999 ▸; Malinowski *et al.*, 1998 ▸; Juszczyk & Karpiński, 2001 ▸). Heterometallic Pd–Re clusters are suitable precursors for such a catalytic system. We found that the reaction of Pd(PPh_3_)_4_ with 1,3-di­methyl­imidazolium-2-carboxyl­ate and Re_2_(CO)_10_ in a toluene–aceto­nitrile mixture produces a mixture of two compounds: [Pd(IMe)_4_][Re_4_(CO)_16_]·2C_4_H_10_O (**1**) and Pd_4_Re_2_(PPh_3_)_4_(μ-CO)_8_(CO)_2_ (**2**) where IMe is 1,3-di­methyl­imidazolium-2-yl­idene. Two other products, tri­phenyl­phosphine oxide and the known complex Re_2_(CO)_8_(PPh_3_)_2_ (Adams *et al.*, 2013 ▸) were isolated from the reaction mixture.
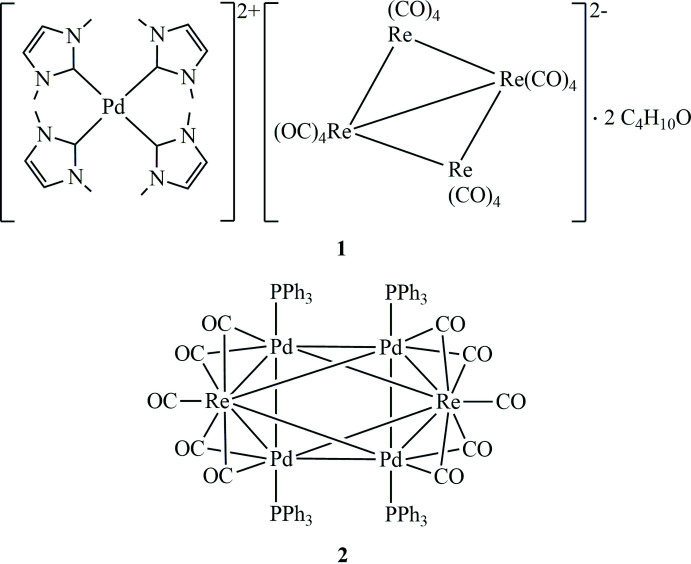


## Structural commentary   

**Figure 1:**
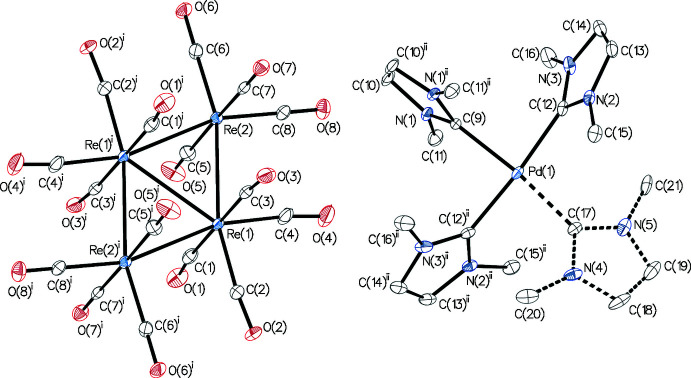
Displacement ellipsoid plot of Pd(IMe)_4_Re_4_(CO)_16_·2C_4_H_10_O (**1**), drawn at the 30% probability level. All hydrogen atoms and solvent mol­ecules are omitted for clarity.

The displacement ellipsoid plot of **1** is depicted in Fig. 1 ▸. The mol­ecular unit of **1** comprises a palladium(II) cation with four coordinated *N*-heterocyclic carbenes (NHC) lying on a twofold rotoinversion axis, and one [Re_4_(CO)_16_] anion. The geometry around the Pd atom is square-planar with one carbene unit being disordered. The C—Pd—C angles range from 86.9 (4) to 97.7 (4)°. The cluster anion lying on the inversion center has a perfectly flat rhombus geometry with the shortest Re—Re bond [2.9767 (3) Å] corresponding to the short diagonal. The other four Re—Re bond lengths [3.001 (2)–3.0132 (2) Å] are also close to double the covalent Re radii (1.51 Å; Cordero *et al.*, 2008 ▸). The Re—Re—Re angles are 59.330 (6)–60.542 (6)°.

**Figure 2:**
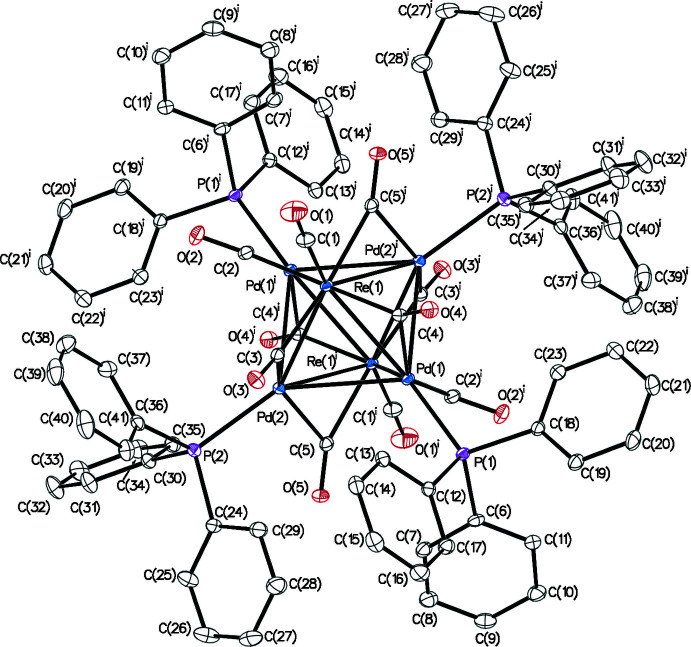
Displacement ellipsoid plot of Pd_4_Re_2_(PPh_3_)_4_(μ-CO)_8_(CO)_2_ (**2**), drawn at the 30% probability level. All hydrogen atoms are omitted for clarity.

The displacement ellipsoid plot of **2** is depicted in Fig. 2 ▸. The geometry of the Re_2_Pd_4_ core is found to be slightly distorted from that of a *D*_4*h*_-symmetric tetra­gonal–bipyramidal prism. In complex **2**, the Pd—Re bond lengths [2.7582 (2)–2.7796 (2) Å] are close to the sum of the covalent Pd and Re radii (1.39 + 1.51 = 2.90 Å). In comparison, the Pd—Re bond lengths in the PdRe_4_(CO)_16_(μ-SbPh_2_)_2_(μ-H)_2_ cluster (Adams *et al.*, 2015 ▸) are in the range 2.9348 (18)–2.9823 (19) Å. The Pd_4_ fragment has an almost square geometry [the Pd—Pd—Pd angles are 89.865 (6)–90.135 (6)° and the Pd—Pd bond lengths are 2.9678 (2)–2.99 (2) Å].

## Supra­molecular features   

**Figure 3:**
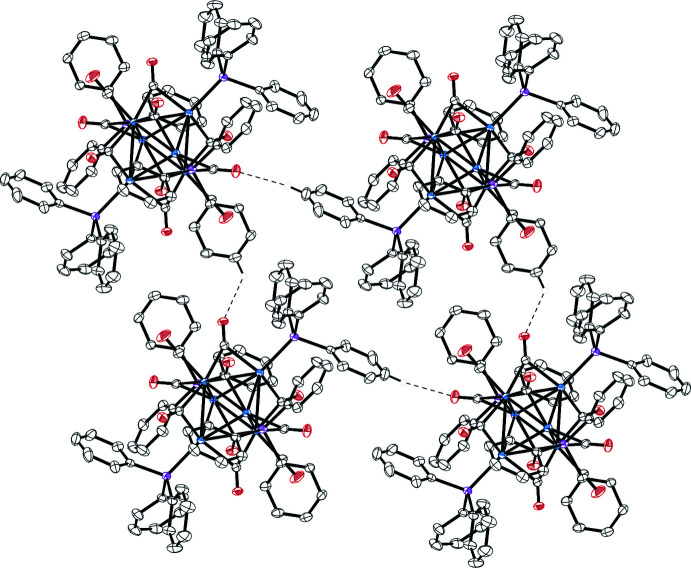
A view of the packing of compound **1**.

**Figure 4:**
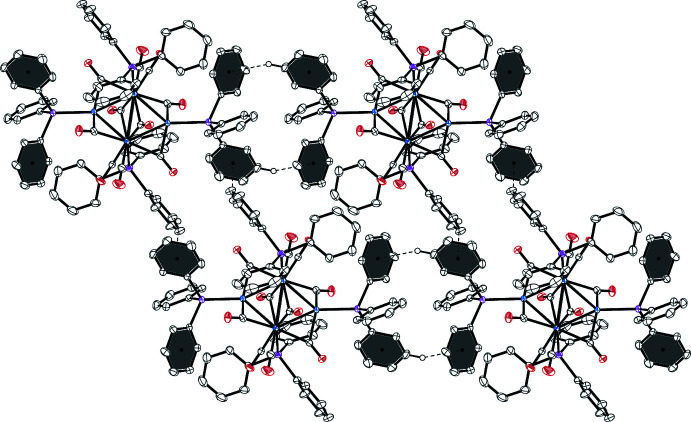
A view of the packing of compound **2**.

**Figure 5:**
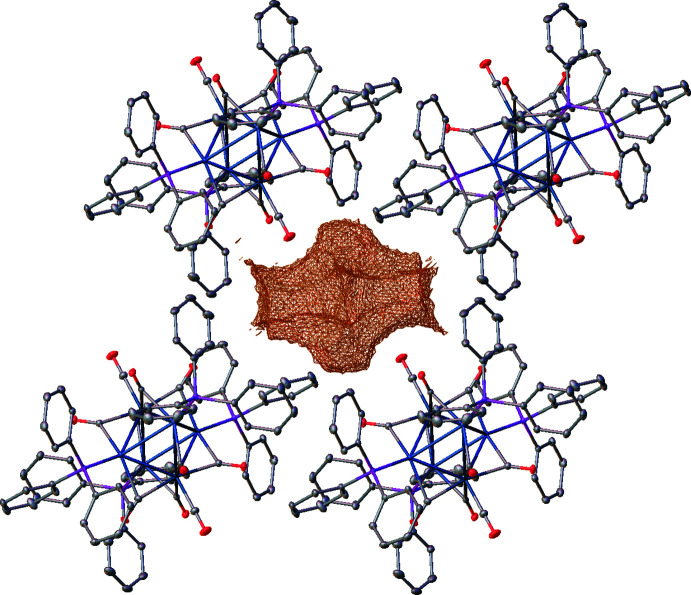
The axial CO groups of the Re(CO)_5_ fragments in **2** point towards voids filled with an unidentified solvent.

In the ionic crystal of **1**, each cation is surrounded by six anions and *vice versa* (Fig. 3 ▸). No classical hydrogen-bonding inter­actions are observed between cations and anions, but many carbonyl-O⋯H_3_C and carbonyl-O⋯HC inter­molecular contacts (Table 1 ▸) are present. The diethyl ether mol­ecule resides in voids between four adjacent cations and anions featuring an O⋯HC contact (2.32 Å) with one of the carbenes at the palladium atom. No π–π stacking is observed in structure **2**, but several weak C—H⋯π and C—H⋯OC contacts (Fig. 4 ▸ and Table 2 ▸) are present. The axial CO groups of the Re(CO)_5_ fragments point towards voids filled with an unidentified solvent (Fig. 5 ▸).

## Database survey   

A search for related structures of palladium cations in the Cambridge Structural Database (CSD Version 5.42, update of November 2020; Groom *et al.*, 2016 ▸) resulted in 27 hits. Of the structures found, the closest structures considering the connectivity of the atoms are tetra­kis­(*N*-methyl­imidazolin-2-yl­idene)palladium(II) diiodide (JOKCIV; Fehlhammer *et al.*, 1992 ▸) and bis­[methyl­enebis(3-methyl­imidazol-2-yl­idene)]palladium(II) diiodide di­methyl­sulfoxide solvate (REFQID; Heckenroth *et al.*, 2006 ▸). The cation in **1** is the first structurally characterized palladium complex ion containing four NHC ligands with substituents at the 1,3 positions of the imidazole ring. There are a number of compounds containing the tetra­nuclear [Re_4_(CO)_16_]^2−^ anion, which is also found in the compound reported here. A search of the CSD found two closely related cluster compounds, **viz**. bis­(tetra­ethyl­ammo­nium) hexa­deca­carbonyl-tetra­rhenium (EAMCRE; Ciani *et al.*, 1978 ▸) and bis­(tetra-*n*-butyl­ammonium)­hexa­deca­carbonyl­tetra­rhenium (BATCRE10; Churchill & Bau, 1968 ▸). The palladium–rhenium carbonyl cluster in **2** has not been structurally characterized previously.

## Synthesis and crystallization   

Under a nitro­gen atmosphere, Pd(PPh_3_)_4_ (241 mg, 0.185 mmol) was added to a toluene–aceto­nitrile mixture (8 and 6 mL, respectively) and 1,3-di­methyl­imidazolium-2-carboxyl­ate (104 mg, 0.704 mmol). The reaction mixture was refluxed for 1.5 h, then Re_2_(CO)_10_ (242 mg, 0.141 mmol) was added, the solution turned dark red and the solvents were removed *in vacuo*. The solid was washed with benzene (3 × 5 ml) and recrystallized from an aceto­nitrile–di­ethyl­ether mixture. X-ray quality crystals of Pd(IMe)_4_Re_4_(CO)_16_·2C_4_H_10_O (37 mg, 13%) were grown from a di­chloro­methane–di­ethyl­ether mixture at 277 K. ^1^HNMR (300.13 MHz, DMSO-*d*_6_, ppm): 3.41 (*s*, 24H, 8Me), 7.37 (*s*, 8H, 8CH). ^13^C{H} NMR (75.4 MHz, DMSO-*d*_6_, ppm): 36.9 (Me, IMe), 123.5 (CH, IMe), 168.0 (C, IMe), 197.7 (CO), 198.7 (CO), 201.1 (CO), 218.6 (CO) IR (ATR, ν, cm^−1^): 3152 (*w*, *br*), 1998 (*vw*), 1974 (*vw*), 1955 (*m*), 1927 (*vw*), 1912 (*vw*), 1881 (*vs*, *br*), 1858 (vw), 1575 (*vw*), 1465 (*w*), 1400 (*vw*), 1332 (*vw*), 1229 (*m*), 1131 (*vw*), 1083 (*vw*), 1013 (*vw*), 845 (*vw*), 736 (*s*), 701 (*vw*), 681 (*m*), 600 (*w*), 577 (*s*), 560 (*vw*), 508 (*vw*), 496 (*vw*), 464 (*w*), 436 (*vw*), 411 (*w*).

A few crystals of Pd_4_Re_2_(PPh_3_)_4_(m-CO)_8_(CO)_2_ suitable for X-ray diffraction analysis were obtained from a yellow benzene solution, after several days, by slow ether diffusion into a concentrated solution of benzene at 277 K. IR (ATR, ν, cm^−1^): 3850 (*vw*), 3054 (*vw*, *br*), 2955 (*vw*, *br*), 1986 (*s*), 1821 (*vs*, *br*), 1585 (*vw*), 1571 (*vw*), 1515 (*vw*), 1477 (*w*), 1434 (*m*), 1307 (*vw*), 1263 (*vw*), 1236 (*vw*, *br*), 1182 (*vw*), 1159 (*vw*), 1119 (*vw*), 1092 (*m*), 1071 (*vw*), 1026 (*vw*), 997 (*w*), 907 (*vw*), 846 (*vw*), 741 (*m*), 690 (*vs*), 618 (*vw*), 565 (*w*), 541 (*vw*), 496 (*m*), 412 (*vw*).

Tri­phenyl­phosphine oxide (14 mg, 28%) and Re_2_(CO)_8_(PPh_3_)_2_ (29 mg, 14%) were also isolated from this crystallization.

## Refinement   

Crystal data, data collection and structure refinement details are summarized in Table 3 ▸. All H atoms were positioned geometrically and refined using a riding model, with C—H = 0.95 Å (*sp* ^2^), 0.98 Å (meth­yl) and 0.99 Å (methyl­ene), with common isotropic temperature factors for all hydrogen atoms of the aromatic rings and methyl groups. SADI restraints on bond lengths and DELU restraints on anisotropic thermal parameters were used to model the disordered carbene ligand and diethyl ether mol­ecule over two positions. For the refinement of **2**, four reflections (100, 010, 200, 0
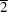
1) were omitted because they showed a significantly lower intensity than calculated, most probably caused by obstruction from the beam stop. The residual electron density in **2** was difficult to model and therefore, the SQUEEZE routine (Spek, 2015 ▸) in *PLATON* (Spek, 2020 ▸) was used to remove the contribution of the electron density in the solvent region from the intensity data and the solvent-free model was employed for the final refinement. The cavity with a volume of *ca* 311 Å^3^ contains approximately 98 electrons.

## Supplementary Material

Crystal structure: contains datablock(s) global, 1, 2. DOI: 10.1107/S2056989021009270/tx2041sup1.cif

Structure factors: contains datablock(s) 1. DOI: 10.1107/S2056989021009270/tx20411sup2.hkl

Structure factors: contains datablock(s) 2. DOI: 10.1107/S2056989021009270/tx20412sup3.hkl

CCDC references: 2108168, 2108167

Additional supporting information:  crystallographic information; 3D view; checkCIF report

## Figures and Tables

**Table 1:** Hydrogen-bond geometry (Å, °) for **1** [Chem scheme1]

*D*—H⋯*A*	*D*—H	H⋯*A*	*D*⋯*A*	*D*—H⋯*A*
C11—H11*A*⋯O4^i^	0.98	2.49	3.436 (6)	161
C13—H13⋯O9^ii^	0.95	2.44	3.36 (3)	165
C13—H13⋯O9*A* ^ii^	0.95	2.32	3.25 (5)	163
C15—H15*A*⋯O6^iii^	0.98	2.44	3.326 (5)	150
C16—H16*B*⋯O9	0.98	2.57	3.49 (3)	158
C18—H18⋯O7^iv^	0.95	2.43	3.230 (9)	141
C19—H19⋯O7^iii^	0.95	2.56	3.483 (16)	163
C20—H20*C*⋯O5^v^	0.98	2.35	3.203 (12)	145
C21—H21*A*⋯O2^vi^	0.98	2.54	3.413 (11)	149
C21—H21*C*⋯O5	0.98	2.59	3.494 (12)	153
C24—H24*B*⋯O8^vii^	0.99	2.58	3.473 (12)	150

Symmetry codes: (i) -x, -y+1, -z; (ii) -x+{\script{1\over 2}}, -y+{\script{3\over 2}}, -z+1; (iii) x, -y+2, z+{\script{1\over 2}}; (iv) -x, -y+2, -z; (v) -x, y, -z+{\script{1\over 2}}; (vi) -x+{\script{1\over 2}}, y+{\script{1\over 2}}, -z+{\script{1\over 2}}; (vii) x+{\script{1\over 2}}, -y+{\script{3\over 2}}, z+{\script{1\over 2}}.

**Table 2:** Hydrogen-bond geometry (Å, °) for **2** [Chem scheme1] *Cg*1 and *Cg*3 are the centroids of the C6–C11 and C18–C23 rings, respectively.

*D*—H⋯*A*	*D*—H	H⋯*A*	*D*⋯*A*	*D*—H⋯*A*
C9—H9⋯O5^i^	0.95	2.49	3.188 (3)	130
C39—H39⋯O2^ii^	0.95	2.60	3.491 (4)	157
C20—H20⋯*Cg*1^iii^	0.95	2.84	3.635 (3)	142
C34—H34⋯*Cg*3^iv^	0.95	2.90	3.683 (3)	140

Symmetry codes: (i) -x+1, -y+1, -z+1; (ii) -x, -y+1, -z+2; (iii) -x+1, -y+2, -z+1; (iv) x, y-1, z.

**Table 3:** Experimental details

	**1**	**2**
Crystal data		
Chemical formula	[Pd(C_5_H_8_N_2_)_4_][Re_4_(CO)_16_]·2C_4_H_10_O	[Pd_4_Re_2_(C_18_H_15_P)_4_(CO)_10_]
*M*_r_	1832.13	2127.18
Crystal system, space group	Monoclinic, *C*2/*c*	Triclinic, *P*\overline{1}
Temperature (K)	100	100
*a*, *b*, *c* (Å)	21.1079 (9), 14.0026 (6), 19.4346 (8)	12.9278 (4), 13.5132 (5), 14.1184 (5)
α, β, γ (°)	90, 109.342 (1), 90	105.983 (1), 108.510 (1), 106.129 (1)
*V* (Å^3^)	5420.0 (4)	2060.09 (12)
*Z*	4	1
Radiation type	Mo *K*α	Mo *K*α
μ (mm^−1^)	9.30	3.91
Crystal size (mm)	0.17 × 0.11 × 0.03	0.23 × 0.18 × 0.18

Data collection		
Diffractometer	Bruker APEXII CCD	Bruker APEXII CCD
Absorption correction	Multi-scan (*SADABS*; Krause *et al.*, 2015 ▸)	Multi-scan (*SADABS*; Krause *et al.*, 2015 ▸)
*T*_min_, *T*_max_	0.285, 0.746	0.515, 0.746
No. of measured, independent and observed [*I* > 2σ(*I*)] reflections	128368, 9046, 7392	151194, 11588, 10906
*R*_int_	0.087	0.042
(sin θ/λ)_max_ (Å^−1^)	0.736	0.696

Refinement		
*R*[*F*^2^ > 2σ(*F* ^2^)], *wR*(*F* ^2^), *S*	0.028, 0.065, 1.06	0.018, 0.042, 1.10
No. of reflections	9046	11588
No. of parameters	427	461
No. of restraints	45	0
H-atom treatment	H-atom parameters constrained	H-atom parameters constrained
Δρ_max_, Δρ_min_ (e Å^−3^)	1.43, −1.83	0.95, −0.70

Computer programs: *APEX2* and *SAINT* (Bruker, 2015 ▸), *SHELXT2014/5* (Sheldrick, 2015*a* ▸), *SHELXL2014/7* (Sheldrick, 2015*b* ▸) and *OLEX2* (Dolomanov *et al.*, 2009 ▸).

## supplementary crystallographic information

### Tetrakis(1,3-dimethylimidazolium-2-ylidene)palladium(II) hexadecacarbonyltetrarhenium diethyl ether disolvate (1) Crystal data

**Table:**

[Pd(C_5_H_8_N_2_)_4_][Re_4_(CO)_16_]·2C_4_H_10_O	*F*(000) = 3448
*M**_r_* = 1832.13	*D*_x_ = 2.245 Mg m^−^^3^
Monoclinic, *C*2/*c*	Mo *K*α radiation, λ = 0.71073 Å
*a* = 21.1079 (9) Å	Cell parameters from 9678 reflections
*b* = 14.0026 (6) Å	θ = 2.9–31.5°
*c* = 19.4346 (8) Å	µ = 9.30 mm^−^^1^
β = 109.342 (1)°	*T* = 100 K
*V* = 5420.0 (4) Å^3^	Plate, brownish yellow
*Z* = 4	0.17 × 0.11 × 0.03 mm

### Tetrakis(1,3-dimethylimidazolium-2-ylidene)palladium(II) hexadecacarbonyltetrarhenium diethyl ether disolvate (1) Data collection

**Table:**

Bruker APEXII CCD diffractometer	7392 reflections with *I* > 2σ(*I*)
φ and ω scans	*R*_int_ = 0.087
Absorption correction: multi-scan (SADABS; Krause *et al.*, 2015)	θ_max_ = 31.5°, θ_min_ = 1.8°
*T*_min_ = 0.285, *T*_max_ = 0.746	*h* = −31→31
128368 measured reflections	*k* = −20→20
9046 independent reflections	*l* = −28→28

### Tetrakis(1,3-dimethylimidazolium-2-ylidene)palladium(II) hexadecacarbonyltetrarhenium diethyl ether disolvate (1) Refinement

**Table:**

Refinement on *F*^2^	Hydrogen site location: inferred from neighbouring sites
Least-squares matrix: full	H-atom parameters constrained
*R*[*F*^2^ > 2σ(*F*^2^)] = 0.028	*w* = 1/[σ^2^(*F*_o_^2^) + (0.0239*P*)^2^ + 18.7547*P*] where *P* = (*F*_o_^2^ + 2*F*_c_^2^)/3
*wR*(*F*^2^) = 0.065	(Δ/σ)_max_ = 0.002
*S* = 1.06	Δρ_max_ = 1.43 e Å^−^^3^
9046 reflections	Δρ_min_ = −1.83 e Å^−^^3^
427 parameters	Extinction correction: SHELXL-2014/7 (Sheldrick, 2015b), Fc^*^=kFc[1+0.001xFc^2^λ^3^/sin(2θ)]^-1/4^
45 restraints	Extinction coefficient: 0.000167 (12)
Primary atom site location: dual	

### Tetrakis(1,3-dimethylimidazolium-2-ylidene)palladium(II) hexadecacarbonyltetrarhenium diethyl ether disolvate (1) Special details

**Table:**

Geometry. All esds (except the esd in the dihedral angle between two l.s. planes) are estimated using the full covariance matrix. The cell esds are taken into account individually in the estimation of esds in distances, angles and torsion angles; correlations between esds in cell parameters are only used when they are defined by crystal symmetry. An approximate (isotropic) treatment of cell esds is used for estimating esds involving l.s. planes.

### Tetrakis(1,3-dimethylimidazolium-2-ylidene)palladium(II) hexadecacarbonyltetrarhenium diethyl ether disolvate (1) Fractional atomic coordinates and isotropic or equivalent isotropic displacement parameters (Å^2^)

**Table:**

	*x*	*y*	*z*	*U*_iso_*/*U*_eq_	Occ. (<1)
Re1	0.24123 (2)	0.64550 (2)	0.00757 (2)	0.01933 (4)	
Re2	0.12119 (2)	0.77460 (2)	−0.02794 (2)	0.01862 (4)	
O1	0.26345 (18)	0.6591 (2)	0.17414 (18)	0.0402 (8)	
O2	0.33004 (14)	0.4665 (2)	0.04457 (16)	0.0282 (6)	
O3	0.21798 (18)	0.6155 (2)	−0.15832 (16)	0.0395 (8)	
O4	0.12446 (17)	0.5083 (3)	−0.0062 (3)	0.0723 (15)	
O5	0.14525 (17)	0.7926 (3)	0.13868 (17)	0.0467 (9)	
O6	0.02877 (15)	0.9489 (2)	−0.05934 (19)	0.0360 (7)	
O7	0.09872 (16)	0.7518 (2)	−0.19407 (16)	0.0298 (6)	
O8	0.00308 (15)	0.6418 (2)	−0.03953 (18)	0.0344 (7)	
C1	0.2553 (2)	0.6594 (3)	0.1126 (2)	0.0259 (8)	
C2	0.30013 (19)	0.5380 (3)	0.0300 (2)	0.0222 (7)	
C3	0.2273 (2)	0.6311 (3)	−0.0977 (2)	0.0265 (8)	
C4	0.1656 (2)	0.5633 (3)	−0.0026 (3)	0.0436 (13)	
C5	0.1390 (2)	0.7867 (3)	0.0783 (2)	0.0284 (8)	
C6	0.06388 (19)	0.8834 (3)	−0.0472 (2)	0.0265 (8)	
C7	0.10950 (19)	0.7595 (3)	−0.1328 (2)	0.0203 (7)	
C8	0.0477 (2)	0.6912 (3)	−0.0359 (2)	0.0240 (7)	
Pd1	0.0000	0.73266 (3)	0.2500	0.01708 (8)	
N1	0.01104 (15)	0.5294 (2)	0.20006 (16)	0.0208 (6)	
N2	0.08671 (16)	0.7599 (2)	0.40944 (16)	0.0207 (6)	
N3	0.14126 (16)	0.6837 (3)	0.35200 (18)	0.0264 (7)	
C9	0.0000	0.5885 (3)	0.2500	0.0163 (9)	
C10	0.0072 (2)	0.4355 (3)	0.2188 (2)	0.0291 (8)	
H10	0.0135	0.3810	0.1927	0.035*	
C11	0.0244 (2)	0.5602 (3)	0.1342 (2)	0.0280 (8)	
H11A	−0.0169	0.5553	0.0921	0.042*	
H11B	0.0591	0.5194	0.1264	0.042*	
H11C	0.0399	0.6266	0.1400	0.042*	
C12	0.08194 (18)	0.7269 (3)	0.34247 (19)	0.0197 (7)	
C13	0.1480 (2)	0.7374 (3)	0.4598 (2)	0.0272 (8)	
H13	0.1630	0.7529	0.5102	0.033*	
C14	0.1825 (2)	0.6895 (3)	0.4242 (2)	0.0306 (9)	
H14	0.2265	0.6643	0.4445	0.037*	
C15	0.0357 (2)	0.8150 (3)	0.4261 (2)	0.0263 (8)	
H15A	0.0515	0.8807	0.4381	0.040*	
H15B	0.0265	0.7864	0.4678	0.040*	
H15C	−0.0055	0.8154	0.3837	0.040*	
C16	0.1617 (2)	0.6371 (4)	0.2951 (3)	0.0372 (11)	
H16A	0.1578	0.5677	0.2988	0.056*	
H16B	0.2084	0.6538	0.3015	0.056*	
H16C	0.1327	0.6587	0.2471	0.056*	
N4	−0.0689 (5)	0.9322 (6)	0.2333 (5)	0.0253 (17)	0.5
N5	0.0364 (4)	0.9444 (6)	0.2548 (5)	0.0250 (16)	0.5
C17	−0.0127 (4)	0.8789 (5)	0.2461 (10)	0.0184 (18)	0.5
C18	−0.0558 (5)	1.0282 (6)	0.2331 (5)	0.038 (2)	0.5
H18	−0.0875	1.0788	0.2239	0.045*	0.5
C19	0.0112 (8)	1.0359 (6)	0.2485 (19)	0.037 (4)	0.5
H19	0.0361	1.0935	0.2541	0.044*	0.5
C20	−0.1373 (5)	0.8957 (8)	0.2173 (6)	0.034 (2)	0.5
H20A	−0.1550	0.8755	0.1662	0.051*	0.5
H20B	−0.1659	0.9462	0.2261	0.051*	0.5
H20C	−0.1367	0.8411	0.2491	0.051*	0.5
C21	0.1067 (6)	0.9243 (7)	0.2723 (6)	0.030 (2)	0.5
H21A	0.1261	0.9091	0.3243	0.045*	0.5
H21B	0.1295	0.9803	0.2612	0.045*	0.5
H21C	0.1126	0.8698	0.2433	0.045*	0.5
O9	0.3252 (14)	0.7269 (9)	0.3616 (17)	0.034 (3)	0.625 (15)
C23	0.3548 (9)	0.5629 (10)	0.3719 (8)	0.038 (3)	0.625 (15)
H23A	0.3132	0.5457	0.3330	0.057*	0.625 (15)
H23B	0.3473	0.5604	0.4191	0.057*	0.625 (15)
H23C	0.3903	0.5177	0.3721	0.057*	0.625 (15)
C22	0.3752 (7)	0.6610 (9)	0.3592 (8)	0.027 (3)	0.625 (15)
H22A	0.4184	0.6774	0.3970	0.033*	0.625 (15)
H22B	0.3815	0.6642	0.3110	0.033*	0.625 (15)
C24	0.3390 (5)	0.8233 (8)	0.3464 (6)	0.032 (2)	0.625 (15)
H24A	0.3523	0.8250	0.3021	0.038*	0.625 (15)
H24B	0.3766	0.8490	0.3877	0.038*	0.625 (15)
C25	0.2780 (5)	0.8827 (7)	0.3352 (5)	0.045 (2)	0.625 (15)
H25A	0.2418	0.8594	0.2924	0.068*	0.625 (15)
H25B	0.2881	0.9493	0.3274	0.068*	0.625 (15)
H25C	0.2639	0.8784	0.3783	0.068*	0.625 (15)
O9A	0.333 (3)	0.7156 (16)	0.371 (3)	0.033 (6)	0.375 (15)
C22A	0.3655 (13)	0.6404 (13)	0.3477 (13)	0.029 (5)	0.375 (15)
H22C	0.4123	0.6592	0.3540	0.035*	0.375 (15)
H22D	0.3422	0.6280	0.2952	0.035*	0.375 (15)
C24A	0.3289 (11)	0.8017 (10)	0.3296 (9)	0.027 (4)	0.375 (15)
H24C	0.2916	0.7966	0.2827	0.032*	0.375 (15)
H24D	0.3712	0.8108	0.3188	0.032*	0.375 (15)
C23A	0.3655 (13)	0.5520 (12)	0.3901 (11)	0.020 (3)	0.375 (15)
H23D	0.3939	0.5037	0.3783	0.030*	0.375 (15)
H23E	0.3195	0.5277	0.3776	0.030*	0.375 (15)
H23F	0.3831	0.5665	0.4424	0.030*	0.375 (15)
C25A	0.3175 (8)	0.8841 (9)	0.3715 (8)	0.037 (4)	0.375 (15)
H25D	0.3118	0.9421	0.3419	0.056*	0.375 (15)
H25E	0.3562	0.8918	0.4161	0.056*	0.375 (15)
H25F	0.2770	0.8731	0.3844	0.056*	0.375 (15)

### Tetrakis(1,3-dimethylimidazolium-2-ylidene)palladium(II) hexadecacarbonyltetrarhenium diethyl ether disolvate (1) Atomic displacement parameters (Å^2^)

**Table:**

	*U*^11^	*U*^22^	*U*^33^	*U*^12^	*U*^13^	*U*^23^
Re1	0.01832 (7)	0.01206 (7)	0.02740 (8)	0.00109 (5)	0.00727 (5)	−0.00045 (5)
Re2	0.01763 (7)	0.01595 (7)	0.02291 (7)	0.00130 (5)	0.00757 (5)	−0.00270 (5)
O1	0.053 (2)	0.039 (2)	0.0373 (17)	0.0041 (16)	0.0264 (16)	0.0060 (14)
O2	0.0337 (15)	0.0177 (14)	0.0362 (15)	0.0062 (12)	0.0156 (12)	0.0079 (12)
O3	0.052 (2)	0.0295 (17)	0.0295 (16)	0.0116 (15)	0.0032 (14)	−0.0019 (13)
O4	0.0295 (17)	0.0229 (19)	0.163 (5)	−0.0023 (14)	0.029 (2)	−0.004 (2)
O5	0.0373 (18)	0.075 (3)	0.0284 (16)	0.0133 (18)	0.0116 (14)	−0.0083 (17)
O6	0.0276 (14)	0.0205 (15)	0.059 (2)	0.0059 (12)	0.0128 (14)	−0.0041 (14)
O7	0.0361 (16)	0.0266 (16)	0.0279 (15)	0.0001 (12)	0.0122 (12)	0.0020 (12)
O8	0.0324 (15)	0.0296 (17)	0.0465 (18)	−0.0084 (13)	0.0202 (14)	−0.0073 (14)
C1	0.0295 (19)	0.0196 (19)	0.035 (2)	0.0015 (15)	0.0191 (17)	0.0027 (16)
C2	0.0258 (17)	0.0211 (19)	0.0217 (16)	−0.0004 (14)	0.0107 (14)	0.0010 (14)
C3	0.0299 (19)	0.0134 (18)	0.032 (2)	0.0042 (15)	0.0052 (16)	−0.0015 (15)
C4	0.026 (2)	0.016 (2)	0.088 (4)	0.0035 (17)	0.018 (2)	0.001 (2)
C5	0.0239 (18)	0.030 (2)	0.032 (2)	0.0046 (16)	0.0101 (16)	−0.0061 (17)
C6	0.0214 (17)	0.023 (2)	0.036 (2)	−0.0010 (15)	0.0117 (16)	−0.0049 (17)
C7	0.0233 (17)	0.0119 (16)	0.0270 (18)	0.0007 (13)	0.0099 (14)	0.0018 (13)
C8	0.0289 (18)	0.0175 (18)	0.0281 (19)	0.0023 (15)	0.0127 (15)	−0.0021 (15)
Pd1	0.02756 (18)	0.00932 (17)	0.01613 (16)	0.000	0.00962 (14)	0.000
N1	0.0257 (15)	0.0122 (14)	0.0230 (14)	−0.0005 (12)	0.0062 (12)	−0.0024 (12)
N2	0.0265 (15)	0.0175 (16)	0.0190 (14)	−0.0068 (12)	0.0085 (12)	−0.0013 (11)
N3	0.0223 (15)	0.0286 (19)	0.0294 (17)	−0.0082 (13)	0.0101 (13)	−0.0123 (14)
C9	0.015 (2)	0.012 (2)	0.020 (2)	0.000	0.0026 (17)	0.000
C10	0.042 (2)	0.0100 (17)	0.033 (2)	0.0016 (16)	0.0093 (18)	−0.0034 (15)
C11	0.036 (2)	0.022 (2)	0.0274 (19)	0.0000 (17)	0.0123 (17)	−0.0041 (16)
C12	0.0253 (17)	0.0152 (17)	0.0201 (16)	−0.0068 (14)	0.0096 (14)	−0.0023 (13)
C13	0.031 (2)	0.026 (2)	0.0219 (17)	−0.0112 (16)	0.0042 (15)	−0.0039 (15)
C14	0.0220 (18)	0.031 (2)	0.034 (2)	−0.0065 (16)	0.0023 (16)	−0.0061 (18)
C15	0.033 (2)	0.026 (2)	0.0236 (18)	−0.0035 (17)	0.0148 (16)	−0.0063 (16)
C16	0.025 (2)	0.047 (3)	0.041 (2)	−0.0039 (19)	0.0137 (18)	−0.019 (2)
N4	0.035 (4)	0.023 (4)	0.021 (4)	0.014 (4)	0.012 (5)	0.004 (3)
N5	0.041 (4)	0.020 (4)	0.019 (4)	−0.005 (3)	0.017 (4)	−0.004 (3)
C17	0.030 (6)	0.015 (3)	0.013 (4)	0.004 (3)	0.011 (7)	0.000 (3)
C18	0.074 (6)	0.019 (4)	0.026 (5)	0.018 (4)	0.024 (5)	0.010 (3)
C19	0.068 (9)	0.016 (3)	0.029 (4)	−0.001 (5)	0.020 (12)	−0.001 (5)
C20	0.039 (5)	0.041 (7)	0.027 (5)	0.018 (4)	0.017 (5)	0.009 (5)
C21	0.043 (5)	0.020 (5)	0.032 (5)	−0.017 (5)	0.018 (6)	−0.003 (4)
O9	0.038 (5)	0.033 (5)	0.037 (9)	−0.011 (5)	0.021 (6)	−0.010 (5)
C23	0.043 (8)	0.046 (5)	0.022 (7)	0.000 (5)	0.008 (6)	0.001 (4)
C22	0.021 (4)	0.036 (5)	0.026 (5)	−0.005 (4)	0.010 (4)	−0.005 (4)
C24	0.035 (4)	0.036 (5)	0.023 (5)	−0.008 (4)	0.006 (4)	−0.006 (4)
C25	0.050 (6)	0.043 (5)	0.038 (5)	0.002 (4)	0.008 (4)	−0.006 (4)
O9A	0.055 (15)	0.022 (5)	0.028 (9)	0.000 (6)	0.022 (10)	0.002 (5)
C22A	0.037 (11)	0.028 (8)	0.019 (8)	0.001 (7)	0.005 (7)	0.003 (7)
C24A	0.044 (9)	0.016 (6)	0.015 (7)	−0.005 (6)	0.002 (6)	−0.006 (4)
C23A	0.023 (7)	0.023 (6)	0.009 (8)	−0.007 (5)	−0.001 (6)	−0.005 (5)
C25A	0.044 (8)	0.024 (6)	0.046 (8)	0.005 (5)	0.016 (7)	−0.006 (5)

### Tetrakis(1,3-dimethylimidazolium-2-ylidene)palladium(II) hexadecacarbonyltetrarhenium diethyl ether disolvate (1) Geometric parameters (Å, º)

**Table:**

Re1—Re1^i^	2.9767 (3)	C16—H16B	0.9800
Re1—Re2^i^	3.0133 (2)	C16—H16C	0.9800
Re1—Re2	3.0011 (2)	N4—C17	1.353 (9)
Re1—C1	1.973 (4)	N4—C18	1.373 (11)
Re1—C2	1.909 (4)	N4—C20	1.464 (11)
Re1—C3	1.978 (4)	N5—C17	1.352 (9)
Re1—C4	1.925 (5)	N5—C19	1.378 (11)
Re2—Re1^i^	3.0134 (2)	N5—C21	1.436 (11)
Re2—C5	1.980 (4)	C18—H18	0.9500
Re2—C6	1.904 (4)	C18—C19	1.349 (15)
Re2—C7	1.982 (4)	C19—H19	0.9500
Re2—C8	1.908 (4)	C20—H20A	0.9800
O1—C1	1.150 (5)	C20—H20B	0.9800
O2—C2	1.167 (5)	C20—H20C	0.9800
O3—C3	1.150 (5)	C21—H21A	0.9800
O4—C4	1.145 (6)	C21—H21B	0.9800
O5—C5	1.139 (5)	C21—H21C	0.9800
O6—C6	1.153 (5)	O9—C22	1.415 (12)
O7—C7	1.140 (5)	O9—C24	1.431 (12)
O8—C8	1.151 (5)	C23—H23A	0.9800
Pd1—C9	2.019 (5)	C23—H23B	0.9800
Pd1—C12^ii^	2.042 (4)	C23—H23C	0.9800
Pd1—C12	2.042 (4)	C23—C22	1.486 (12)
Pd1—C17	2.064 (6)	C22—H22A	0.9900
N1—C9	1.353 (4)	C22—H22B	0.9900
N1—C10	1.375 (5)	C24—H24A	0.9900
N1—C11	1.464 (5)	C24—H24B	0.9900
N2—C12	1.353 (4)	C24—C25	1.488 (12)
N2—C13	1.375 (5)	C25—H25A	0.9800
N2—C15	1.446 (5)	C25—H25B	0.9800
N3—C12	1.347 (5)	C25—H25C	0.9800
N3—C14	1.387 (5)	O9A—C22A	1.413 (16)
N3—C16	1.467 (5)	O9A—C24A	1.439 (16)
C9—N1^ii^	1.353 (4)	C22A—H22C	0.9900
C10—C10^ii^	1.343 (8)	C22A—H22D	0.9900
C10—H10	0.9500	C22A—C23A	1.487 (15)
C11—H11A	0.9800	C24A—H24C	0.9900
C11—H11B	0.9800	C24A—H24D	0.9900
C11—H11C	0.9800	C24A—C25A	1.479 (14)
C13—H13	0.9500	C23A—H23D	0.9800
C13—C14	1.338 (6)	C23A—H23E	0.9800
C14—H14	0.9500	C23A—H23F	0.9800
C15—H15A	0.9800	C25A—H25D	0.9800
C15—H15B	0.9800	C25A—H25E	0.9800
C15—H15C	0.9800	C25A—H25F	0.9800
C16—H16A	0.9800		
			
Re1^i^—Re1—Re2	60.542 (6)	H15B—C15—H15C	109.5
Re1^i^—Re1—Re2^i^	60.127 (5)	N3—C16—H16A	109.5
Re2—Re1—Re2^i^	120.669 (6)	N3—C16—H16B	109.5
C1—Re1—Re1^i^	91.41 (12)	N3—C16—H16C	109.5
C1—Re1—Re2^i^	91.11 (12)	H16A—C16—H16B	109.5
C1—Re1—Re2	90.28 (12)	H16A—C16—H16C	109.5
C1—Re1—C3	179.77 (18)	H16B—C16—H16C	109.5
C2—Re1—Re1^i^	134.85 (11)	C17—N4—C18	112.1 (9)
C2—Re1—Re2	164.59 (11)	C17—N4—C20	126.2 (8)
C2—Re1—Re2^i^	74.73 (11)	C18—N4—C20	121.7 (9)
C2—Re1—C1	88.73 (16)	C17—N5—C19	111.2 (8)
C2—Re1—C3	91.05 (15)	C17—N5—C21	125.8 (8)
C2—Re1—C4	90.39 (17)	C19—N5—C21	122.9 (9)
C3—Re1—Re1^i^	88.79 (11)	N4—C17—Pd1	130.5 (6)
C3—Re1—Re2	89.91 (11)	N5—C17—Pd1	125.7 (6)
C3—Re1—Re2^i^	88.89 (12)	N5—C17—N4	103.8 (8)
C4—Re1—Re1^i^	134.75 (13)	N4—C18—H18	127.0
C4—Re1—Re2^i^	165.04 (13)	C19—C18—N4	105.9 (9)
C4—Re1—Re2	74.23 (13)	C19—C18—H18	127.0
C4—Re1—C1	90.0 (2)	N5—C19—H19	126.5
C4—Re1—C3	89.9 (2)	C18—C19—N5	107.0 (9)
Re1—Re2—Re1^i^	59.330 (6)	C18—C19—H19	126.5
C5—Re2—Re1^i^	89.40 (12)	N4—C20—H20A	109.5
C5—Re2—Re1	87.57 (12)	N4—C20—H20B	109.5
C5—Re2—C7	176.24 (15)	N4—C20—H20C	109.5
C6—Re2—Re1	163.81 (12)	H20A—C20—H20B	109.5
C6—Re2—Re1^i^	104.51 (12)	H20A—C20—H20C	109.5
C6—Re2—C5	91.43 (17)	H20B—C20—H20C	109.5
C6—Re2—C7	91.67 (16)	N5—C21—H21A	109.5
C6—Re2—C8	91.55 (16)	N5—C21—H21B	109.5
C7—Re2—Re1	88.86 (10)	N5—C21—H21C	109.5
C7—Re2—Re1^i^	87.76 (11)	H21A—C21—H21B	109.5
C8—Re2—Re1^i^	163.93 (12)	H21A—C21—H21C	109.5
C8—Re2—Re1	104.61 (12)	H21B—C21—H21C	109.5
C8—Re2—C5	90.28 (17)	C22—O9—C24	113.7 (13)
C8—Re2—C7	91.76 (15)	H23A—C23—H23B	109.5
O1—C1—Re1	174.2 (4)	H23A—C23—H23C	109.5
O2—C2—Re1	172.3 (3)	H23B—C23—H23C	109.5
O3—C3—Re1	174.7 (4)	C22—C23—H23A	109.5
O4—C4—Re1	174.2 (4)	C22—C23—H23B	109.5
O5—C5—Re2	175.9 (4)	C22—C23—H23C	109.5
O6—C6—Re2	179.4 (4)	O9—C22—C23	109.7 (11)
O7—C7—Re2	175.8 (3)	O9—C22—H22A	109.7
O8—C8—Re2	178.7 (4)	O9—C22—H22B	109.7
C9—Pd1—C12	87.72 (10)	C23—C22—H22A	109.7
C9—Pd1—C12^ii^	87.72 (10)	C23—C22—H22B	109.7
C9—Pd1—C17	173.0 (2)	H22A—C22—H22B	108.2
C12^ii^—Pd1—C12	175.4 (2)	O9—C24—H24A	109.8
C12^ii^—Pd1—C17	86.9 (4)	O9—C24—H24B	109.8
C12—Pd1—C17	97.7 (4)	O9—C24—C25	109.4 (11)
C9—N1—C10	110.8 (3)	H24A—C24—H24B	108.2
C9—N1—C11	125.2 (3)	C25—C24—H24A	109.8
C10—N1—C11	124.0 (3)	C25—C24—H24B	109.8
C12—N2—C13	111.0 (3)	C24—C25—H25A	109.5
C12—N2—C15	125.0 (3)	C24—C25—H25B	109.5
C13—N2—C15	123.9 (3)	C24—C25—H25C	109.5
C12—N3—C14	110.9 (3)	H25A—C25—H25B	109.5
C12—N3—C16	126.1 (3)	H25A—C25—H25C	109.5
C14—N3—C16	123.1 (4)	H25B—C25—H25C	109.5
N1^ii^—C9—Pd1	127.7 (2)	C22A—O9A—C24A	113 (2)
N1—C9—Pd1	127.7 (2)	O9A—C22A—H22C	109.5
N1—C9—N1^ii^	104.6 (4)	O9A—C22A—H22D	109.5
N1—C10—H10	126.6	O9A—C22A—C23A	110.6 (16)
C10^ii^—C10—N1	106.9 (2)	H22C—C22A—H22D	108.1
C10^ii^—C10—H10	126.6	C23A—C22A—H22C	109.5
N1—C11—H11A	109.5	C23A—C22A—H22D	109.5
N1—C11—H11B	109.5	O9A—C24A—H24C	109.8
N1—C11—H11C	109.5	O9A—C24A—H24D	109.8
H11A—C11—H11B	109.5	O9A—C24A—C25A	109.5 (18)
H11A—C11—H11C	109.5	H24C—C24A—H24D	108.2
H11B—C11—H11C	109.5	C25A—C24A—H24C	109.8
N2—C12—Pd1	127.2 (3)	C25A—C24A—H24D	109.8
N3—C12—Pd1	128.1 (3)	C22A—C23A—H23D	109.5
N3—C12—N2	104.6 (3)	C22A—C23A—H23E	109.5
N2—C13—H13	126.5	C22A—C23A—H23F	109.5
C14—C13—N2	107.0 (3)	H23D—C23A—H23E	109.5
C14—C13—H13	126.5	H23D—C23A—H23F	109.5
N3—C14—H14	126.7	H23E—C23A—H23F	109.5
C13—C14—N3	106.6 (4)	C24A—C25A—H25D	109.5
C13—C14—H14	126.7	C24A—C25A—H25E	109.5
N2—C15—H15A	109.5	C24A—C25A—H25F	109.5
N2—C15—H15B	109.5	H25D—C25A—H25E	109.5
N2—C15—H15C	109.5	H25D—C25A—H25F	109.5
H15A—C15—H15B	109.5	H25E—C25A—H25F	109.5
H15A—C15—H15C	109.5		
			
N2—C13—C14—N3	0.1 (5)	C16—N3—C14—C13	179.1 (4)
C9—N1—C10—C10^ii^	0.6 (6)	N4—C18—C19—N5	2 (3)
C10—N1—C9—Pd1	179.8 (2)	C17—N4—C18—C19	−2 (2)
C10—N1—C9—N1^ii^	−0.2 (2)	C17—N5—C19—C18	−2 (3)
C11—N1—C9—Pd1	−1.0 (4)	C18—N4—C17—Pd1	−178.0 (11)
C11—N1—C9—N1^ii^	179.0 (4)	C18—N4—C17—N5	0.7 (17)
C11—N1—C10—C10^ii^	−178.6 (4)	C19—N5—C17—Pd1	179.6 (18)
C12—N2—C13—C14	0.0 (5)	C19—N5—C17—N4	1 (2)
C12—N3—C14—C13	−0.3 (5)	C20—N4—C17—Pd1	−1 (2)
C13—N2—C12—Pd1	175.6 (3)	C20—N4—C17—N5	177.6 (9)
C13—N2—C12—N3	−0.2 (4)	C20—N4—C18—C19	−179.0 (17)
C14—N3—C12—Pd1	−175.4 (3)	C21—N5—C17—Pd1	−4 (2)
C14—N3—C12—N2	0.3 (4)	C21—N5—C17—N4	177.6 (9)
C15—N2—C12—Pd1	−6.8 (5)	C21—N5—C19—C18	−179.0 (14)
C15—N2—C12—N3	177.5 (3)	C22—O9—C24—C25	168.3 (18)
C15—N2—C13—C14	−177.7 (4)	C24—O9—C22—C23	−177.2 (18)
C16—N3—C12—Pd1	5.2 (6)	C22A—O9A—C24A—C25A	−160 (3)
C16—N3—C12—N2	−179.1 (4)	C24A—O9A—C22A—C23A	−177 (3)

Symmetry codes: (i) −*x*+1/2, −*y*+3/2, −*z*; (ii) −*x*, *y*, −*z*+1/2.

### Tetrakis(1,3-dimethylimidazolium-2-ylidene)palladium(II) hexadecacarbonyltetrarhenium diethyl ether disolvate (1) Hydrogen-bond geometry (Å, º)

**Table:**

*D*—H···*A*	*D*—H	H···*A*	*D*···*A*	*D*—H···*A*
C11—H11*A*···O4^iii^	0.98	2.49	3.436 (6)	161
C13—H13···O9^iv^	0.95	2.44	3.36 (3)	165
C13—H13···O9*A*^iv^	0.95	2.32	3.25 (5)	163
C15—H15*A*···O6^v^	0.98	2.44	3.326 (5)	150
C16—H16*B*···O9	0.98	2.57	3.49 (3)	158
C18—H18···O7^vi^	0.95	2.43	3.230 (9)	141
C19—H19···O7^v^	0.95	2.56	3.483 (16)	163
C20—H20*C*···O5^ii^	0.98	2.35	3.203 (12)	145
C21—H21*A*···O2^vii^	0.98	2.54	3.413 (11)	149
C21—H21*C*···O5	0.98	2.59	3.494 (12)	153
C24—H24*B*···O8^viii^	0.99	2.58	3.473 (12)	150

Symmetry codes: (ii) −*x*, *y*, −*z*+1/2; (iii) −*x*, −*y*+1, −*z*; (iv) −*x*+1/2, −*y*+3/2, −*z*+1; (v) *x*, −*y*+2, *z*+1/2; (vi) −*x*, −*y*+2, −*z*; (vii) −*x*+1/2, *y*+1/2, −*z*+1/2; (viii) *x*+1/2, −*y*+3/2, *z*+1/2.

### Octa-µ-carbonyl-dicarbonyltetrakis(triphenylphosphane)palladiumdirhenium (2) Crystal data

**Table:**

[Pd_4_Re_2_(C_18_H_15_P)_4_(CO)_10_]	*Z* = 1
*M**_r_* = 2127.18	*F*(000) = 1026
Triclinic, *P*1	*D*_x_ = 1.715 Mg m^−^^3^
*a* = 12.9278 (4) Å	Mo *K*α radiation, λ = 0.71073 Å
*b* = 13.5132 (5) Å	Cell parameters from 9325 reflections
*c* = 14.1184 (5) Å	θ = 2.8–29.6°
α = 105.983 (1)°	µ = 3.91 mm^−^^1^
β = 108.510 (1)°	*T* = 100 K
γ = 106.129 (1)°	Block, red
*V* = 2060.09 (12) Å^3^	0.23 × 0.18 × 0.18 mm

### Octa-µ-carbonyl-dicarbonyltetrakis(triphenylphosphane)palladiumdirhenium (2) Data collection

**Table:**

Bruker APEXII CCD diffractometer	10906 reflections with *I* > 2σ(*I*)
φ and ω scans	*R*_int_ = 0.042
Absorption correction: multi-scan (SADABS; Krause *et al.*, 2015)	θ_max_ = 29.7°, θ_min_ = 2.8°
*T*_min_ = 0.515, *T*_max_ = 0.746	*h* = −17→17
151194 measured reflections	*k* = −18→18
11588 independent reflections	*l* = −19→19

### Octa-µ-carbonyl-dicarbonyltetrakis(triphenylphosphane)palladiumdirhenium (2) Refinement

**Table:**

Refinement on *F*^2^	Hydrogen site location: inferred from neighbouring sites
Least-squares matrix: full	H-atom parameters constrained
*R*[*F*^2^ > 2σ(*F*^2^)] = 0.018	*w* = 1/[σ^2^(*F*_o_^2^) + (0.0148*P*)^2^ + 2.1161*P*] where *P* = (*F*_o_^2^ + 2*F*_c_^2^)/3
*wR*(*F*^2^) = 0.042	(Δ/σ)_max_ = 0.005
*S* = 1.10	Δρ_max_ = 0.95 e Å^−^^3^
11588 reflections	Δρ_min_ = −0.70 e Å^−^^3^
461 parameters	Extinction correction: SHELXL-2014/7 (Sheldrick 2015b), Fc^*^=kFc[1+0.001xFc^2^λ^3^/sin(2θ)]^-1/4^
0 restraints	Extinction coefficient: 0.00119 (9)
Primary atom site location: dual	

### Octa-µ-carbonyl-dicarbonyltetrakis(triphenylphosphane)palladiumdirhenium (2) Special details

**Table:**

Geometry. All esds (except the esd in the dihedral angle between two l.s. planes) are estimated using the full covariance matrix. The cell esds are taken into account individually in the estimation of esds in distances, angles and torsion angles; correlations between esds in cell parameters are only used when they are defined by crystal symmetry. An approximate (isotropic) treatment of cell esds is used for estimating esds involving l.s. planes.

### Octa-µ-carbonyl-dicarbonyltetrakis(triphenylphosphane)palladiumdirhenium (2) Fractional atomic coordinates and isotropic or equivalent isotropic displacement parameters (Å^2^)

**Table:**

	*x*	*y*	*z*	*U*_iso_*/*U*_eq_	
Re1	−0.03789 (2)	0.58284 (2)	0.59592 (2)	0.01288 (3)	
Pd1	0.11939 (2)	0.64837 (2)	0.50923 (2)	0.01377 (3)	
Pd2	0.13798 (2)	0.50124 (2)	0.63029 (2)	0.01360 (3)	
P1	0.27753 (4)	0.79978 (4)	0.52970 (4)	0.01537 (9)	
P2	0.27280 (5)	0.48255 (4)	0.77255 (4)	0.01634 (10)	
O1	−0.11990 (18)	0.71938 (18)	0.74578 (16)	0.0433 (5)	
O2	−0.12746 (15)	0.42760 (14)	0.70708 (13)	0.0266 (3)	
O3	0.18975 (14)	0.70585 (13)	0.81880 (12)	0.0236 (3)	
O4	0.03880 (14)	0.82425 (13)	0.59258 (13)	0.0240 (3)	
O5	0.30356 (13)	0.45945 (14)	0.53745 (13)	0.0236 (3)	
C1	−0.0874 (2)	0.66930 (19)	0.69145 (18)	0.0246 (4)	
C2	−0.10429 (18)	0.45891 (18)	0.64488 (17)	0.0198 (4)	
C3	0.12010 (18)	0.64157 (17)	0.73175 (16)	0.0178 (4)	
C4	0.03254 (17)	0.73328 (17)	0.57964 (16)	0.0175 (4)	
C5	0.20750 (18)	0.45438 (17)	0.51815 (16)	0.0174 (4)	
C6	0.37893 (17)	0.75557 (17)	0.48099 (17)	0.0188 (4)	
C7	0.4316 (2)	0.69504 (19)	0.53169 (19)	0.0242 (4)	
H7	0.4156	0.6822	0.5894	0.029*	
C8	0.5067 (2)	0.65387 (19)	0.4984 (2)	0.0270 (5)	
H8	0.5409	0.6118	0.5322	0.032*	
C9	0.5315 (2)	0.6744 (2)	0.41544 (19)	0.0283 (5)	
H9	0.5830	0.6464	0.3924	0.034*	
C10	0.4819 (2)	0.7353 (2)	0.36630 (19)	0.0278 (5)	
H10	0.5008	0.7504	0.3106	0.033*	
C11	0.40389 (18)	0.77520 (18)	0.39750 (17)	0.0215 (4)	
H11	0.3683	0.8154	0.3619	0.026*	
C12	0.38172 (18)	0.90224 (17)	0.66794 (16)	0.0180 (4)	
C13	0.34659 (19)	0.91169 (18)	0.75257 (17)	0.0216 (4)	
H13	0.2686	0.8649	0.7381	0.026*	
C14	0.4253 (2)	0.9896 (2)	0.85853 (18)	0.0264 (5)	
H14	0.4004	0.9956	0.9156	0.032*	
C15	0.5392 (2)	1.0578 (2)	0.88081 (19)	0.0299 (5)	
H15	0.5922	1.1113	0.9527	0.036*	
C16	0.5758 (2)	1.0476 (2)	0.7971 (2)	0.0292 (5)	
H16	0.6544	1.0935	0.8122	0.035*	
C17	0.49795 (19)	0.97081 (18)	0.69209 (18)	0.0230 (4)	
H17	0.5237	0.9645	0.6356	0.028*	
C18	0.22924 (18)	0.88071 (17)	0.45395 (16)	0.0184 (4)	
C19	0.3060 (2)	0.98359 (18)	0.46489 (18)	0.0229 (4)	
H19	0.3875	1.0152	0.5148	0.027*	
C20	0.2634 (2)	1.0399 (2)	0.4029 (2)	0.0281 (5)	
H20	0.3162	1.1093	0.4102	0.034*	
C21	0.1449 (2)	0.9955 (2)	0.3312 (2)	0.0347 (6)	
H21	0.1161	1.0346	0.2897	0.042*	
C22	0.0676 (2)	0.8933 (3)	0.3198 (2)	0.0387 (6)	
H22	−0.0139	0.8622	0.2699	0.046*	
C23	0.1098 (2)	0.8368 (2)	0.3815 (2)	0.0282 (5)	
H23	0.0566	0.7676	0.3742	0.034*	
C24	0.42622 (18)	0.57138 (19)	0.80973 (17)	0.0208 (4)	
C25	0.5207 (2)	0.5376 (2)	0.8357 (2)	0.0294 (5)	
H25	0.5070	0.4651	0.8366	0.035*	
C26	0.6346 (2)	0.6093 (2)	0.8602 (2)	0.0401 (6)	
H26	0.6982	0.5855	0.8774	0.048*	
C27	0.6561 (2)	0.7155 (3)	0.8598 (2)	0.0409 (6)	
H27	0.7342	0.7642	0.8765	0.049*	
C28	0.5630 (2)	0.7502 (3)	0.8347 (3)	0.0415 (7)	
H28	0.5776	0.8233	0.8353	0.050*	
C29	0.4486 (2)	0.6779 (2)	0.8087 (2)	0.0326 (5)	
H29	0.3850	0.7015	0.7901	0.039*	
C30	0.26705 (19)	0.34177 (18)	0.75156 (17)	0.0199 (4)	
C31	0.3181 (2)	0.3138 (2)	0.83814 (19)	0.0296 (5)	
H31	0.3604	0.3699	0.9103	0.036*	
C32	0.3073 (3)	0.2043 (2)	0.8192 (2)	0.0365 (6)	
H32	0.3425	0.1860	0.8784	0.044*	
C33	0.2453 (2)	0.1214 (2)	0.7143 (2)	0.0335 (5)	
H33	0.2382	0.0465	0.7018	0.040*	
C34	0.1937 (2)	0.1477 (2)	0.6279 (2)	0.0280 (5)	
H34	0.1506	0.0909	0.5561	0.034*	
C35	0.20507 (19)	0.25749 (18)	0.64649 (17)	0.0213 (4)	
H35	0.1702	0.2754	0.5869	0.026*	
C36	0.25498 (19)	0.52324 (18)	0.89861 (16)	0.0201 (4)	
C37	0.1507 (2)	0.4569 (2)	0.8988 (2)	0.0303 (5)	
H37	0.0943	0.3913	0.8359	0.036*	
C38	0.1295 (3)	0.4865 (3)	0.9907 (2)	0.0414 (7)	
H38	0.0592	0.4403	0.9910	0.050*	
C39	0.2099 (3)	0.5825 (3)	1.0819 (2)	0.0443 (7)	
H39	0.1948	0.6030	1.1446	0.053*	
C40	0.3120 (3)	0.6484 (2)	1.0815 (2)	0.0407 (7)	
H40	0.3671	0.7148	1.1441	0.049*	
C41	0.3356 (2)	0.6192 (2)	0.99088 (18)	0.0277 (5)	
H41	0.4070	0.6649	0.9920	0.033*	

### Octa-µ-carbonyl-dicarbonyltetrakis(triphenylphosphane)palladiumdirhenium (2) Atomic displacement parameters (Å^2^)

**Table:**

	*U*^11^	*U*^22^	*U*^33^	*U*^12^	*U*^13^	*U*^23^
Re1	0.01245 (4)	0.01357 (4)	0.01647 (4)	0.00659 (3)	0.00863 (3)	0.00710 (3)
Pd1	0.01249 (7)	0.01311 (7)	0.01861 (7)	0.00468 (5)	0.00858 (5)	0.00854 (5)
Pd2	0.01236 (7)	0.01464 (7)	0.01648 (7)	0.00662 (5)	0.00670 (5)	0.00807 (5)
P1	0.0134 (2)	0.0141 (2)	0.0215 (2)	0.00506 (18)	0.00935 (18)	0.00943 (19)
P2	0.0163 (2)	0.0175 (2)	0.0174 (2)	0.00879 (19)	0.00679 (18)	0.00836 (19)
O1	0.0443 (11)	0.0454 (11)	0.0439 (11)	0.0246 (9)	0.0282 (9)	0.0054 (9)
O2	0.0308 (8)	0.0263 (8)	0.0236 (7)	0.0053 (7)	0.0156 (7)	0.0124 (6)
O3	0.0248 (8)	0.0196 (7)	0.0221 (7)	0.0100 (6)	0.0058 (6)	0.0062 (6)
O4	0.0241 (8)	0.0162 (7)	0.0341 (8)	0.0097 (6)	0.0128 (7)	0.0111 (6)
O5	0.0180 (7)	0.0314 (8)	0.0300 (8)	0.0145 (6)	0.0135 (6)	0.0156 (7)
C1	0.0216 (10)	0.0260 (11)	0.0263 (10)	0.0104 (9)	0.0120 (9)	0.0075 (9)
C2	0.0168 (9)	0.0221 (10)	0.0220 (9)	0.0069 (8)	0.0104 (8)	0.0090 (8)
C3	0.0190 (9)	0.0182 (9)	0.0213 (9)	0.0089 (8)	0.0109 (8)	0.0109 (8)
C4	0.0138 (8)	0.0190 (9)	0.0199 (9)	0.0070 (7)	0.0074 (7)	0.0073 (7)
C5	0.0190 (9)	0.0161 (9)	0.0218 (9)	0.0082 (7)	0.0109 (8)	0.0101 (7)
C6	0.0142 (9)	0.0151 (9)	0.0270 (10)	0.0041 (7)	0.0108 (8)	0.0079 (8)
C7	0.0234 (10)	0.0231 (10)	0.0361 (12)	0.0112 (9)	0.0180 (9)	0.0177 (9)
C8	0.0252 (11)	0.0224 (11)	0.0411 (13)	0.0132 (9)	0.0172 (10)	0.0163 (10)
C9	0.0222 (11)	0.0284 (12)	0.0331 (12)	0.0127 (9)	0.0131 (9)	0.0063 (9)
C10	0.0257 (11)	0.0367 (13)	0.0256 (10)	0.0144 (10)	0.0157 (9)	0.0113 (9)
C11	0.0174 (9)	0.0242 (10)	0.0233 (10)	0.0077 (8)	0.0092 (8)	0.0104 (8)
C12	0.0175 (9)	0.0154 (9)	0.0226 (9)	0.0069 (7)	0.0079 (8)	0.0100 (7)
C13	0.0216 (10)	0.0218 (10)	0.0255 (10)	0.0108 (8)	0.0098 (8)	0.0132 (8)
C14	0.0305 (12)	0.0291 (11)	0.0236 (10)	0.0146 (10)	0.0112 (9)	0.0139 (9)
C15	0.0316 (12)	0.0213 (11)	0.0251 (11)	0.0071 (9)	0.0031 (9)	0.0071 (9)
C16	0.0219 (11)	0.0220 (11)	0.0326 (12)	0.0024 (9)	0.0058 (9)	0.0089 (9)
C17	0.0201 (10)	0.0196 (10)	0.0287 (10)	0.0060 (8)	0.0104 (8)	0.0110 (8)
C18	0.0200 (9)	0.0191 (9)	0.0228 (9)	0.0097 (8)	0.0122 (8)	0.0120 (8)
C19	0.0243 (10)	0.0201 (10)	0.0284 (10)	0.0082 (8)	0.0143 (9)	0.0126 (8)
C20	0.0370 (13)	0.0227 (11)	0.0358 (12)	0.0133 (10)	0.0215 (10)	0.0190 (10)
C21	0.0403 (14)	0.0394 (14)	0.0441 (14)	0.0236 (12)	0.0217 (12)	0.0318 (12)
C22	0.0243 (12)	0.0525 (17)	0.0478 (15)	0.0158 (12)	0.0116 (11)	0.0357 (14)
C23	0.0199 (10)	0.0316 (12)	0.0356 (12)	0.0069 (9)	0.0106 (9)	0.0219 (10)
C24	0.0159 (9)	0.0252 (10)	0.0209 (9)	0.0081 (8)	0.0070 (8)	0.0097 (8)
C25	0.0229 (11)	0.0282 (12)	0.0347 (12)	0.0144 (9)	0.0076 (9)	0.0103 (10)
C26	0.0217 (12)	0.0435 (15)	0.0497 (16)	0.0166 (11)	0.0105 (11)	0.0130 (13)
C27	0.0200 (11)	0.0465 (16)	0.0527 (16)	0.0072 (11)	0.0150 (11)	0.0218 (14)
C28	0.0262 (13)	0.0395 (15)	0.0630 (18)	0.0097 (11)	0.0167 (12)	0.0322 (14)
C29	0.0216 (11)	0.0350 (13)	0.0496 (15)	0.0127 (10)	0.0150 (11)	0.0278 (12)
C30	0.0208 (10)	0.0204 (10)	0.0236 (10)	0.0118 (8)	0.0100 (8)	0.0115 (8)
C31	0.0352 (13)	0.0260 (11)	0.0273 (11)	0.0163 (10)	0.0071 (10)	0.0134 (9)
C32	0.0438 (15)	0.0329 (13)	0.0403 (14)	0.0248 (12)	0.0120 (12)	0.0223 (11)
C33	0.0376 (13)	0.0249 (12)	0.0465 (14)	0.0204 (11)	0.0179 (12)	0.0183 (11)
C34	0.0304 (12)	0.0219 (11)	0.0335 (12)	0.0132 (9)	0.0152 (10)	0.0087 (9)
C35	0.0213 (10)	0.0202 (10)	0.0238 (10)	0.0097 (8)	0.0098 (8)	0.0093 (8)
C36	0.0226 (10)	0.0250 (10)	0.0195 (9)	0.0154 (8)	0.0091 (8)	0.0120 (8)
C37	0.0246 (11)	0.0393 (14)	0.0319 (12)	0.0155 (10)	0.0143 (10)	0.0157 (10)
C38	0.0430 (16)	0.0624 (19)	0.0491 (16)	0.0326 (15)	0.0350 (14)	0.0348 (15)
C39	0.076 (2)	0.0585 (19)	0.0360 (14)	0.0490 (18)	0.0393 (15)	0.0307 (14)
C40	0.068 (2)	0.0340 (14)	0.0219 (11)	0.0274 (14)	0.0168 (12)	0.0101 (10)
C41	0.0364 (13)	0.0240 (11)	0.0215 (10)	0.0124 (10)	0.0105 (9)	0.0094 (9)

### Octa-µ-carbonyl-dicarbonyltetrakis(triphenylphosphane)palladiumdirhenium (2) Geometric parameters (Å, º)

**Table:**

Re1—Pd1^i^	2.7748 (2)	C15—H15	0.9500
Re1—Pd1	2.7555 (2)	C15—C16	1.394 (4)
Re1—Pd2^i^	2.7796 (2)	C16—H16	0.9500
Re1—Pd2	2.7582 (2)	C16—C17	1.383 (3)
Re1—C1	1.921 (2)	C17—H17	0.9500
Re1—C2	2.058 (2)	C18—C19	1.400 (3)
Re1—C3	2.062 (2)	C18—C23	1.391 (3)
Re1—C4	2.092 (2)	C19—H19	0.9500
Re1—C5^i^	2.087 (2)	C19—C20	1.393 (3)
Pd1—Re1^i^	2.7747 (2)	C20—H20	0.9500
Pd1—Pd2	2.9678 (2)	C20—C21	1.379 (4)
Pd1—Pd2^i^	2.9909 (2)	C21—H21	0.9500
Pd1—P1	2.3291 (5)	C21—C22	1.393 (4)
Pd1—C2^i^	2.170 (2)	C22—H22	0.9500
Pd1—C4	2.088 (2)	C22—C23	1.392 (3)
Pd2—Re1^i^	2.7796 (2)	C23—H23	0.9500
Pd2—Pd1^i^	2.9910 (2)	C24—C25	1.398 (3)
Pd2—P2	2.3317 (5)	C24—C29	1.393 (3)
Pd2—C3	2.158 (2)	C25—H25	0.9500
Pd2—C5	2.094 (2)	C25—C26	1.387 (4)
P1—C6	1.825 (2)	C26—H26	0.9500
P1—C12	1.830 (2)	C26—C27	1.385 (4)
P1—C18	1.825 (2)	C27—H27	0.9500
P2—C24	1.822 (2)	C27—C28	1.389 (4)
P2—C30	1.819 (2)	C28—H28	0.9500
P2—C36	1.821 (2)	C28—C29	1.390 (3)
O1—C1	1.140 (3)	C29—H29	0.9500
O2—C2	1.158 (3)	C30—C31	1.398 (3)
O3—C3	1.162 (2)	C30—C35	1.396 (3)
O4—C4	1.167 (3)	C31—H31	0.9500
O5—C5	1.161 (2)	C31—C32	1.386 (3)
C2—Pd1^i^	2.170 (2)	C32—H32	0.9500
C5—Re1^i^	2.087 (2)	C32—C33	1.387 (4)
C6—C7	1.403 (3)	C33—H33	0.9500
C6—C11	1.389 (3)	C33—C34	1.383 (4)
C7—H7	0.9500	C34—H34	0.9500
C7—C8	1.385 (3)	C34—C35	1.390 (3)
C8—H8	0.9500	C35—H35	0.9500
C8—C9	1.385 (3)	C36—C37	1.398 (3)
C9—H9	0.9500	C36—C41	1.386 (3)
C9—C10	1.376 (3)	C37—H37	0.9500
C10—H10	0.9500	C37—C38	1.386 (4)
C10—C11	1.398 (3)	C38—H38	0.9500
C11—H11	0.9500	C38—C39	1.379 (5)
C12—C13	1.394 (3)	C39—H39	0.9500
C12—C17	1.400 (3)	C39—C40	1.374 (5)
C13—H13	0.9500	C40—H40	0.9500
C13—C14	1.397 (3)	C40—C41	1.386 (3)
C14—H14	0.9500	C41—H41	0.9500
C14—C15	1.381 (3)		
			
Pd1—Re1—Pd1^i^	99.105 (5)	C6—C7—H7	119.7
Pd1—Re1—Pd2^i^	65.413 (5)	C8—C7—C6	120.6 (2)
Pd1^i^—Re1—Pd2^i^	64.596 (5)	C8—C7—H7	119.7
Pd1—Re1—Pd2	65.131 (5)	C7—C8—H8	120.2
Pd2—Re1—Pd1^i^	65.445 (5)	C7—C8—C9	119.6 (2)
Pd2—Re1—Pd2^i^	99.238 (5)	C9—C8—H8	120.2
C1—Re1—Pd1	131.04 (7)	C8—C9—H9	119.9
C1—Re1—Pd1^i^	129.69 (7)	C10—C9—C8	120.3 (2)
C1—Re1—Pd2	133.48 (7)	C10—C9—H9	119.9
C1—Re1—Pd2^i^	127.28 (7)	C9—C10—H10	119.7
C1—Re1—C2	83.99 (9)	C9—C10—C11	120.7 (2)
C1—Re1—C3	85.84 (9)	C11—C10—H10	119.7
C1—Re1—C4	83.85 (9)	C6—C11—C10	119.5 (2)
C1—Re1—C5^i^	81.63 (9)	C6—C11—H11	120.3
C2—Re1—Pd1	141.37 (6)	C10—C11—H11	120.3
C2—Re1—Pd1^i^	50.76 (6)	C13—C12—P1	119.88 (16)
C2—Re1—Pd2	78.91 (6)	C13—C12—C17	118.49 (19)
C2—Re1—Pd2^i^	109.51 (6)	C17—C12—P1	121.62 (16)
C2—Re1—C3	87.43 (8)	C12—C13—H13	119.8
C2—Re1—C4	166.84 (8)	C12—C13—C14	120.4 (2)
C2—Re1—C5^i^	91.78 (8)	C14—C13—H13	119.8
C3—Re1—Pd1^i^	109.34 (6)	C13—C14—H14	119.8
C3—Re1—Pd1	80.75 (6)	C15—C14—C13	120.4 (2)
C3—Re1—Pd2	50.73 (6)	C15—C14—H14	119.8
C3—Re1—Pd2^i^	143.08 (6)	C14—C15—H15	120.2
C3—Re1—C4	86.69 (8)	C14—C15—C16	119.6 (2)
C3—Re1—C5^i^	167.46 (8)	C16—C15—H15	120.2
C4—Re1—Pd1	48.71 (5)	C15—C16—H16	119.9
C4—Re1—Pd1^i^	142.40 (5)	C17—C16—C15	120.2 (2)
C4—Re1—Pd2^i^	82.00 (5)	C17—C16—H16	119.9
C4—Re1—Pd2	106.14 (5)	C12—C17—H17	119.5
C5^i^—Re1—Pd1	107.18 (6)	C16—C17—C12	120.9 (2)
C5^i^—Re1—Pd1^i^	79.46 (5)	C16—C17—H17	119.5
C5^i^—Re1—Pd2	141.24 (5)	C19—C18—P1	123.40 (16)
C5^i^—Re1—Pd2^i^	48.43 (5)	C23—C18—P1	117.73 (16)
C5^i^—Re1—C4	91.42 (8)	C23—C18—C19	118.9 (2)
Re1—Pd1—Re1^i^	80.894 (5)	C18—C19—H19	119.8
Re1^i^—Pd1—Pd2	57.781 (5)	C20—C19—C18	120.3 (2)
Re1^i^—Pd1—Pd2^i^	57.011 (4)	C20—C19—H19	119.8
Re1—Pd1—Pd2	57.477 (4)	C19—C20—H20	119.8
Re1—Pd1—Pd2^i^	57.681 (5)	C21—C20—C19	120.3 (2)
Pd2—Pd1—Pd2^i^	90.135 (6)	C21—C20—H20	119.8
P1—Pd1—Re1^i^	133.608 (14)	C20—C21—H21	120.0
P1—Pd1—Re1	143.861 (14)	C20—C21—C22	119.9 (2)
P1—Pd1—Pd2^i^	143.699 (14)	C22—C21—H21	120.0
P1—Pd1—Pd2	125.624 (14)	C21—C22—H22	120.0
C2^i^—Pd1—Re1^i^	47.26 (6)	C23—C22—C21	119.9 (2)
C2^i^—Pd1—Re1	122.58 (6)	C23—C22—H22	120.0
C2^i^—Pd1—Pd2^i^	72.07 (5)	C18—C23—C22	120.6 (2)
C2^i^—Pd1—Pd2	100.12 (6)	C18—C23—H23	119.7
C2^i^—Pd1—P1	93.27 (6)	C22—C23—H23	119.7
C4—Pd1—Re1	48.82 (6)	C25—C24—P2	123.95 (18)
C4—Pd1—Re1^i^	125.79 (6)	C29—C24—P2	117.27 (17)
C4—Pd1—Pd2	99.39 (6)	C29—C24—C25	118.8 (2)
C4—Pd1—Pd2^i^	76.94 (5)	C24—C25—H25	119.8
C4—Pd1—P1	100.27 (6)	C26—C25—C24	120.3 (2)
C4—Pd1—C2^i^	143.15 (8)	C26—C25—H25	119.8
Re1—Pd2—Re1^i^	80.762 (5)	C25—C26—H26	119.8
Re1—Pd2—Pd1^i^	57.545 (5)	C27—C26—C25	120.5 (2)
Re1^i^—Pd2—Pd1	57.623 (5)	C27—C26—H26	119.8
Re1—Pd2—Pd1	57.392 (5)	C26—C27—H27	120.2
Re1^i^—Pd2—Pd1^i^	56.904 (4)	C26—C27—C28	119.7 (2)
Pd1—Pd2—Pd1^i^	89.865 (6)	C28—C27—H27	120.2
P2—Pd2—Re1^i^	139.454 (14)	C27—C28—H28	120.0
P2—Pd2—Re1	138.742 (14)	C27—C28—C29	119.9 (3)
P2—Pd2—Pd1^i^	128.138 (15)	C29—C28—H28	120.0
P2—Pd2—Pd1	141.995 (15)	C24—C29—H29	119.6
C3—Pd2—Re1	47.70 (5)	C28—C29—C24	120.8 (2)
C3—Pd2—Re1^i^	123.96 (5)	C28—C29—H29	119.6
C3—Pd2—Pd1^i^	99.53 (5)	C31—C30—P2	122.07 (17)
C3—Pd2—Pd1	74.39 (5)	C35—C30—P2	119.19 (16)
C3—Pd2—P2	96.08 (6)	C35—C30—C31	118.7 (2)
C5—Pd2—Re1	124.34 (6)	C30—C31—H31	119.9
C5—Pd2—Re1^i^	48.21 (6)	C32—C31—C30	120.3 (2)
C5—Pd2—Pd1^i^	99.26 (6)	C32—C31—H31	119.9
C5—Pd2—Pd1	74.81 (6)	C31—C32—H32	119.8
C5—Pd2—P2	96.36 (6)	C31—C32—C33	120.5 (2)
C5—Pd2—C3	143.64 (8)	C33—C32—H32	119.8
C6—P1—Pd1	112.32 (7)	C32—C33—H33	120.0
C6—P1—C12	100.69 (9)	C34—C33—C32	119.9 (2)
C12—P1—Pd1	117.97 (7)	C34—C33—H33	120.0
C18—P1—Pd1	112.89 (7)	C33—C34—H34	120.1
C18—P1—C6	105.65 (10)	C33—C34—C35	119.8 (2)
C18—P1—C12	105.98 (9)	C35—C34—H34	120.1
C24—P2—Pd2	111.89 (7)	C30—C35—H35	119.6
C30—P2—Pd2	116.83 (7)	C34—C35—C30	120.9 (2)
C30—P2—C24	106.51 (10)	C34—C35—H35	119.6
C30—P2—C36	101.65 (10)	C37—C36—P2	117.43 (17)
C36—P2—Pd2	114.10 (7)	C41—C36—P2	123.41 (18)
C36—P2—C24	104.65 (10)	C41—C36—C37	119.1 (2)
O1—C1—Re1	178.1 (2)	C36—C37—H37	119.9
Re1—C2—Pd1^i^	81.98 (7)	C38—C37—C36	120.1 (2)
O2—C2—Re1	152.98 (18)	C38—C37—H37	119.9
O2—C2—Pd1^i^	124.90 (17)	C37—C38—H38	119.8
Re1—C3—Pd2	81.57 (7)	C39—C38—C37	120.4 (3)
O3—C3—Re1	152.27 (17)	C39—C38—H38	119.8
O3—C3—Pd2	126.08 (16)	C38—C39—H39	120.2
Pd1—C4—Re1	82.47 (7)	C40—C39—C38	119.6 (2)
O4—C4—Re1	149.43 (17)	C40—C39—H39	120.2
O4—C4—Pd1	128.10 (16)	C39—C40—H40	119.6
Re1^i^—C5—Pd2	83.36 (7)	C39—C40—C41	120.8 (3)
O5—C5—Re1^i^	149.52 (17)	C41—C40—H40	119.6
O5—C5—Pd2	127.10 (16)	C36—C41—C40	120.0 (3)
C7—C6—P1	115.78 (16)	C36—C41—H41	120.0
C11—C6—P1	124.87 (17)	C40—C41—H41	120.0
C11—C6—C7	119.32 (19)		
			
Pd1—P1—C6—C7	−58.98 (17)	C15—C16—C17—C12	0.0 (4)
Pd1—P1—C6—C11	119.34 (17)	C17—C12—C13—C14	1.2 (3)
Pd1—P1—C12—C13	−21.58 (19)	C18—P1—C6—C7	177.54 (16)
Pd1—P1—C12—C17	157.12 (15)	C18—P1—C6—C11	−4.1 (2)
Pd1—P1—C18—C19	171.11 (16)	C18—P1—C12—C13	106.03 (18)
Pd1—P1—C18—C23	−9.1 (2)	C18—P1—C12—C17	−75.28 (19)
Pd2—P2—C24—C25	−139.46 (18)	C18—C19—C20—C21	0.8 (4)
Pd2—P2—C24—C29	38.6 (2)	C19—C18—C23—C22	0.9 (4)
Pd2—P2—C30—C31	−162.18 (17)	C19—C20—C21—C22	−0.6 (4)
Pd2—P2—C30—C35	14.8 (2)	C20—C21—C22—C23	0.6 (4)
Pd2—P2—C36—C37	66.91 (19)	C21—C22—C23—C18	−0.8 (4)
Pd2—P2—C36—C41	−109.26 (18)	C23—C18—C19—C20	−0.9 (3)
P1—C6—C7—C8	177.66 (18)	C24—P2—C30—C31	72.0 (2)
P1—C6—C11—C10	−178.91 (17)	C24—P2—C30—C35	−111.09 (18)
P1—C12—C13—C14	179.96 (17)	C24—P2—C36—C37	−170.48 (18)
P1—C12—C17—C16	−179.77 (18)	C24—P2—C36—C41	13.4 (2)
P1—C18—C19—C20	178.85 (17)	C24—C25—C26—C27	0.3 (4)
P1—C18—C23—C22	−178.9 (2)	C25—C24—C29—C28	−1.0 (4)
P2—C24—C25—C26	178.2 (2)	C25—C26—C27—C28	0.1 (5)
P2—C24—C29—C28	−179.2 (2)	C26—C27—C28—C29	−0.9 (5)
P2—C30—C31—C32	177.2 (2)	C27—C28—C29—C24	1.4 (5)
P2—C30—C35—C34	−176.80 (17)	C29—C24—C25—C26	0.1 (4)
P2—C36—C37—C38	−177.0 (2)	C30—P2—C24—C25	−10.7 (2)
P2—C36—C41—C40	175.83 (19)	C30—P2—C24—C29	167.39 (19)
C6—P1—C12—C13	−144.12 (17)	C30—P2—C36—C37	−59.76 (19)
C6—P1—C12—C17	34.58 (19)	C30—P2—C36—C41	124.07 (19)
C6—P1—C18—C19	−65.8 (2)	C30—C31—C32—C33	−0.3 (4)
C6—P1—C18—C23	114.00 (18)	C31—C30—C35—C34	0.2 (3)
C6—C7—C8—C9	1.1 (4)	C31—C32—C33—C34	−0.1 (4)
C7—C6—C11—C10	−0.6 (3)	C32—C33—C34—C35	0.5 (4)
C7—C8—C9—C10	0.0 (4)	C33—C34—C35—C30	−0.6 (4)
C8—C9—C10—C11	−1.4 (4)	C35—C30—C31—C32	0.2 (4)
C9—C10—C11—C6	1.7 (3)	C36—P2—C24—C25	96.5 (2)
C11—C6—C7—C8	−0.8 (3)	C36—P2—C24—C29	−85.4 (2)
C12—P1—C6—C7	67.42 (18)	C36—P2—C30—C31	−37.3 (2)
C12—P1—C6—C11	−114.25 (19)	C36—P2—C30—C35	139.62 (18)
C12—P1—C18—C19	40.5 (2)	C36—C37—C38—C39	1.1 (4)
C12—P1—C18—C23	−139.70 (18)	C37—C36—C41—C40	−0.3 (3)
C12—C13—C14—C15	−0.3 (3)	C37—C38—C39—C40	−0.6 (4)
C13—C12—C17—C16	−1.1 (3)	C38—C39—C40—C41	−0.4 (4)
C13—C14—C15—C16	−0.8 (4)	C39—C40—C41—C36	0.8 (4)
C14—C15—C16—C17	1.0 (4)	C41—C36—C37—C38	−0.7 (4)

Symmetry code: (i) −*x*, −*y*+1, −*z*+1.

### Octa-µ-carbonyl-dicarbonyltetrakis(triphenylphosphane)palladiumdirhenium (2) Hydrogen-bond geometry (Å, º)

*Cg*1 and *Cg*3 are the centroids of the C6–C11 and C18–C23 rings, respectively.

**Table:**

*D*—H···*A*	*D*—H	H···*A*	*D*···*A*	*D*—H···*A*
C9—H9···O5^ii^	0.95	2.49	3.188 (3)	130
C39—H39···O2^iii^	0.95	2.60	3.491 (4)	157
C20—H20···*Cg*1^iv^	0.95	2.84	3.635 (3)	142
C34—H34···*Cg*3^v^	0.95	2.90	3.683 (3)	140

Symmetry codes: (ii) −*x*+1, −*y*+1, −*z*+1; (iii) −*x*, −*y*+1, −*z*+2; (iv) −*x*+1, −*y*+2, −*z*+1; (v) *x*, *y*−1, *z*.
